# Stimulator of interferon genes (STING) immunohistochemical expression in fumarate hydratase-deficient renal cell carcinoma: biological and potential predictive implications

**DOI:** 10.1007/s00428-025-04041-5

**Published:** 2025-02-03

**Authors:** S. Marletta, L. Marcolini, A. Caliò, S. Pedron, P. Antonini, F. M. Martelli, L. Stefanizzi, G. Martignoni

**Affiliations:** 1 Department of Diagnostic and Public Health, Section of Pathology, University of Verona, Largo L. Scuro 10, 37134 Verona, Italy; 2 Division of Pathology, Humanitas Istituto Clinico Catanese, Catania, Italy; 3 Department of Pathology, Pederzoli Hospital, Peschiera del Garda, Italy

**Keywords:** Fumarate hydratase-deficient renal cell carcinoma, STING, 2SC, PD-L1

## Abstract

Fumarate hydratase (FH)-deficient renal cell carcinoma is an aggressive neoplasm driven by inactivating mutations of the *FH* gene, which cause metabolites like S-(2-succinyl)cysteine (2SC) to accumulate and trigger cascades supporting malignant transformation. Although in preclinical models the c-GAS-STING pathway is activated by fumarate metabolites, its role in humans has not been explored yet. Eleven FH-deficient renal cell carcinomas, including primary neoplasms and metastases, were retrieved and evaluated for clinical-pathological features and immunohistochemical expression of FH, 2SC (commercially available), and STING. The in-house collection accounted for 0.2% of the 2011–2023 renal cell carcinomas cohort (5/2210). Eight-on-ten cases with available follow-up behaved aggressively (local recurrence/distant metastases). All tumors revealed FH staining loss and strong and diffuse 2SC immunolabeling. At least focal STING expression was detected in most primary tumors (9/11, 82%), often (78%) in a wide percentage of cells (≥ 30%). Notably, significant STING expression was observed in all but two aggressive renal neoplasms, with one of the remaining showing increased staining in its hepatic localization, and in 86% (6/7) of neoplasms significantly expressing PD-L1. In our series, (i) FH-deficient renal cell carcinoma represents 0.2% of in-house cases; (ii) combining FH loss and positive 2SC staining now commercially available is useful in primary and secondary tumors, supporting this latter marker’s safe routine adoption; and (iii) a significant STING labeling (≥ 30%) in most of the samples, especially in those behaving aggressively and expressing PD-L1, provides novel insights regarding the molecular basis of FH-deficient renal cell carcinomas, proposing STING as a potential predictive marker.

## Introduction

Fumarate hydratase (FH)-deficient renal cell carcinoma is a rare renal cell neoplasm characterized by broad histological heterogeneity [1], both in primary tumors [2–4] and in secondary localizations [5]. A dismal prognosis is usually accustomed to these tumors, with most patients rapidly developing local recurrences or distant metastases [6, 7]. Molecularly, such neoplasms are driven by germline or somatic (20% of the cases) inactivating mutations of the *FH* gene [6, 8]. The former encodes for a metabolic enzyme physiologically involved in the mitochondrial conversion of fumarate to malate within the Krebs tricarboxylic acids cycle. In FH-deficient tumors, the loss of FH activity leads to aberrant accumulation of fumarate [9] and products of its reaction with the thiol groups of free cysteine and proteins, generating S-(2-succinyl)cysteine (2SC) and succinated proteins, respectively [10]. Immunohistochemistry-based protein detection techniques may exploit this so-called “succination” process relying on 2SC antibodies, which could be thus adopted as a surrogate of FH-loss. However, such assays have been mainly employed for research purposes [11], while only recently the utility of a commercially available specific 2SC antibody has been documented in routine diagnostic of FH-deficient renal cell carcinoma in a single study [12].

Regarding tumorigenesis, the metabolites linked to FH-loss may trigger several downstream cascades that ultimately support malignant transformation, including enhanced glycolytic activity [13], increased *EGFR* [14] and *VEGF* [15] transcription, and chronic inflammation [16]. Recently, two elegant studies in preclinical models have shown aberrant fumarate metabolites may cause mitochondrial vesicles containing DNA (mtDNA) to be released in the cytoplasm. In detail, the succination of the outer mitochondrial membrane proteins [17] or the alteration of its electrical potential [18] likely causes a progressive remodeling of mitochondrial morphology, characterized by swollen and elongation of mitochondria. This latter may favor mtDNA extrusion to the cytoplasm through mitochondrial-derived vesicles regulated by the endocytic accessory protein SNX9 [19]. Then, once in the cytosol, mtDNA might activate the c-GAS-STING pathway, which elicits a cellular inflammatory interferon (IFN) I-based phenotype, proven by upregulation of pro-inflammatory cytokines and chemokines in a TBK1-dependent manner [16]. Physiologically responsible for the response to intracellular double-stranded DNA fragments in the autophagy process [20] and in the immune reaction to viruses [21], alterations of the stimulator of interferon genes (STING) pathway have been linked to the pathogenesis of both autoimmune disorders [22] and cancer [23]. As for this latter, an IFN-I signature is generally retained as a marker of an immunologically “hot” tumor. Accordingly, an immune infiltration is detected in a variable proportion of FH-deficient renal cell carcinomas [24], although the underlying pathological basis of such a finding has been understood so far. Despite the cGAS-STING pathway having been studied in the aforementioned preclinical models, it has not been explored in a clinical series of FH-deficient renal cell carcinoma. Thus, in the present work, we sought to investigate the immunohistochemical expression of STING in eleven FH-deficient renal cell carcinomas and to correlate it with histological features, 2SC immunostaining, tumor inflammatory infiltrate, and potential clinical implications.

## Methods

### Patients and samples

Eleven cases of FH-deficient renal cell carcinomas were retrieved from the files of participant institutions, including samples from both primary neoplasms and distant metastases when available. In this series, we have added two unreported cases and included nine of eleven of the previously described cases [4, 5] due to the availability of material suitable for further analyses. Five patients were from the entire collection of in-house renal cell carcinoma cases (Pederzoli Hospital) and six were consultations. All procedures performed in our study involving human participants received approval (Prog. 4136CESC) and were in accordance with the ethical standards of the institutional and/or national research committee and with the declaration of Helsinki. Clinical data on a previous history of other renal tumors in relatives and of personal extrarenal neoplasm was also recorded. All slides were reviewed by four authors (A.C., S.M., L.M., and G.M.). Each neoplasm was evaluated for its clinical outcome and pathological features. The distribution of tumor-infiltrating lymphocytes (TILs) was described in our previous report [5] and other acknowledged studies [25, 26]: “desert” (no TIL), “excluded” (TILs just gathering around the lesion without significant infiltration within the tumor), and “inflamed” (TILs intermixed with the tumor cells in the whole neoplastic area).

### Immunohistochemistry

Sections from tissue blocks of all tumors were immunohistochemically stained with FH (clone J-13 Santa Cruz Biotech, dilution 1:50; DIAPATH), 2SC (clone CRB2005017_3, dilution 1:2000, Biosynth), STING (anti-TMEM173; clone SP338, dilution 1:150; Abcam, UK), and PD-1 (clone CAL-20, prediluted; ROCHE). Heat-induced antigen retrieval for STING was performed using a microwave oven and 0.01 mol/L of citrate buffer, pH 8.0, for 30 min. Moreover, tumors defined as “excluded” and “inflamed” by hematoxylin and eosin based on referral classifications [25, 26] were investigated for the specific composition of different lymphocytes subpopulations, according to the different percentages of expression of the following antibodies: CD3 (clone PS1, dilution 1:200, LEICA), CD20 (clone L26, prediluted, NOVO-CASTRA), CD4 (clone 4B12, dilution 1.150, LEICA), CD8 (clone 29S, dilution 1:20, LEICA), and GATA 3 (clone L50-823, dilution 1:150; BD Pharmingen, USA). All samples were processed using a sensitive “Bond Polymer Refine” detection system in an automated Bond immunohistochemistry instrument (Leica Biosystems, Germany). The most commonly used immunohistochemical assay developed to determine PD-L1 expression level was performed (clone SP263, prediluted; ROCHE) on the Ventana Benchmark Ultra platform according to the manufacturer's instructions. Membranous immune expression of PD-L1 on neoplastic cells was recorded as the percentage of positive neoplastic elements. The appropriate positive (tonsil and placenta) and negative controls were concurrently performed.

## Results

### Clinical and pathological features

Among the eleven patients, the five belonging to the in-house collection accounted for 0.2% of the whole cohort of renal cell carcinomas from 2011 to 2023 (5/2210). The clinical and pathological characteristics of nine of the eleven included patients have already been described [5]. Thus, data from the whole series are globally summarized below, while the specific features of the two novel added cases are detailed in the following separated subsections for completeness.

Four patients were females, while seven were males (F: M ratio 1:1.75), ranging from 33 to 66 years old (mean 50, median 44). Lesions ranged in size from 4.3 to 9 cm (mean 6.1, median 5.7). Morphologically, various and often admixed architectural growth patterns were detected, spanning from papillary to tubule-glandular, tubule-cystic, alveolar, solid, and sieve-like. Hardly all tumors were classified as high-grade (G3-G4) (10/11, 91%), including six G3 and four G4 cases, three of these latter revealing sarcomatoid dedifferentiation (27%, cases 7, 8, and 9). The remaining renal lesion (case 3) was entirely composed of low-grade (G2) eosinophilic alveolar-arranged tumor cells, strikingly resembling succinate dehydrogenase-deficient renal cell carcinoma. As mentioned in our previous series [5], according to its immunohistochemical findings, it was then diagnosed as a low-grade oncocytic FH-deficient renal cell carcinoma.

Follow-up was available for ten patients, ranging from 2 to 77 months (mean 30 months, median 39 months), eight of whom (8/10, 80%) experienced aggressive clinical behavior developing either local recurrence (2/10, 20%), distant metastases (7/10, 70%), or both (1/10, 10%). Secondary metastases variably involved abdominal lymph nodes (4/10, 40%), the liver (4/10, 40%), the peritoneum (3/10, 30%), the lungs (2/10, 20%), and the abdomen wall soft tissues (1/10, 10%). Among them, three distant metastases from two patients were histologically documented, respectively involving the peritoneum (case 1), the liver (case 6), and an abdominal node (case 6).

#### Case 10

**Fig. 1:**
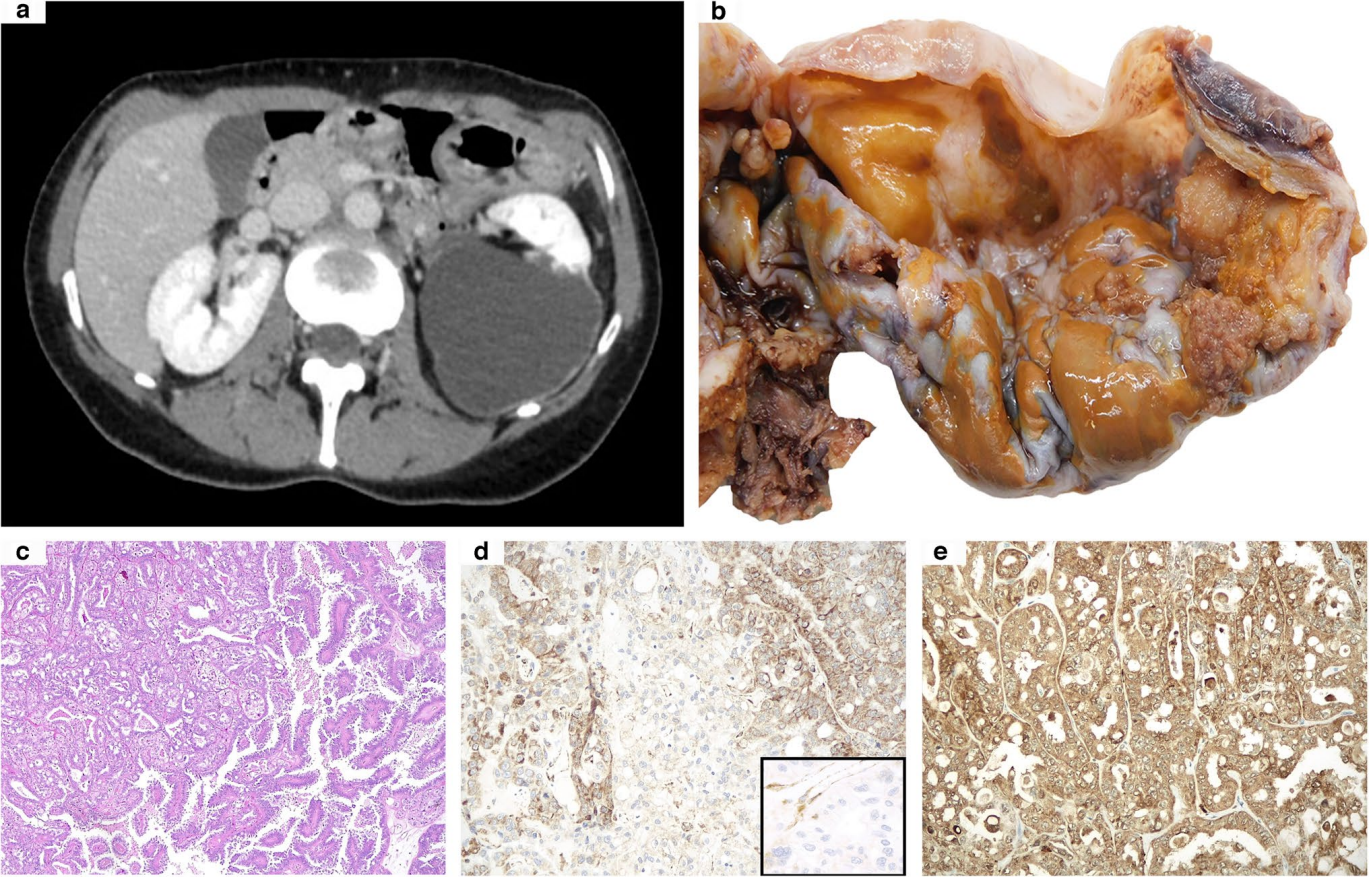
Abdominal CT scan from case 10 revealing a 7-cm predominantly cystic left renal mass (**a**). At gross examination, the inner cystic wall was partially filled with brownish papillary-looking protrusions (**b**). Morphologically, papillary and tubule-glandular architectural growth patterns were closely admixed (**c**). Immunohistochemical analysis showed a patchy FH loss: while some tumor areas partially retained its expression, others revealed clear-cut negativity with positive endothelial cells serving as internal control (**d** and inset). However, strong and diffuse 2SC staining supported the diagnosis of FH-deficient renal cell carcinoma (**e**) (original magnifications 50X (**c**), 200X (**d**, **e**), and 400X inset)

A 47-year-old woman, who had previously undergone hysterectomy for uterine leiomyomas, received partial nephrectomy for a single cystic mass of the left kidney, classified as Bosniak IV at preoperative radiological imaging (Fig. 1a). Grossly, the surgical specimen was composed of a 6.5-cm predominantly cystic lesion with several brownish papillary-like areas in the inner surface (Fig. 1b). At histological examination, a neoplastic proliferation arranged in various architectural growth patterns was observed (Fig. 1c), including papillary (80%), tubule-glandular (10%), solid (5%), and sieve-like (5%). Tumor cells showed abundant eosinophilic cytoplasm and roundish nuclei with prominent nucleoli (G3 sec. ISUP/WHO 2022). Coagulative tumoral necrosis was variably spotted. On follow-up, multiple peritoneal nodules were discovered, so that she was given a combined therapy based on an anti-angiogenetic drug (cabozantinib) and an immune-checkpoint inhibitor (nivolumab), still ongoing at the time of the present manuscript. Genetic tests were carried out based on the clinical and pathological findings, revealing a germline alteration of the *FH* gene (c.698G > A). Moreover, an identical mutation of the same gene was also identified in one of the patient’s sons, further confirming the underlying hereditary syndrome.

#### Case 11

**Fig. 2:**
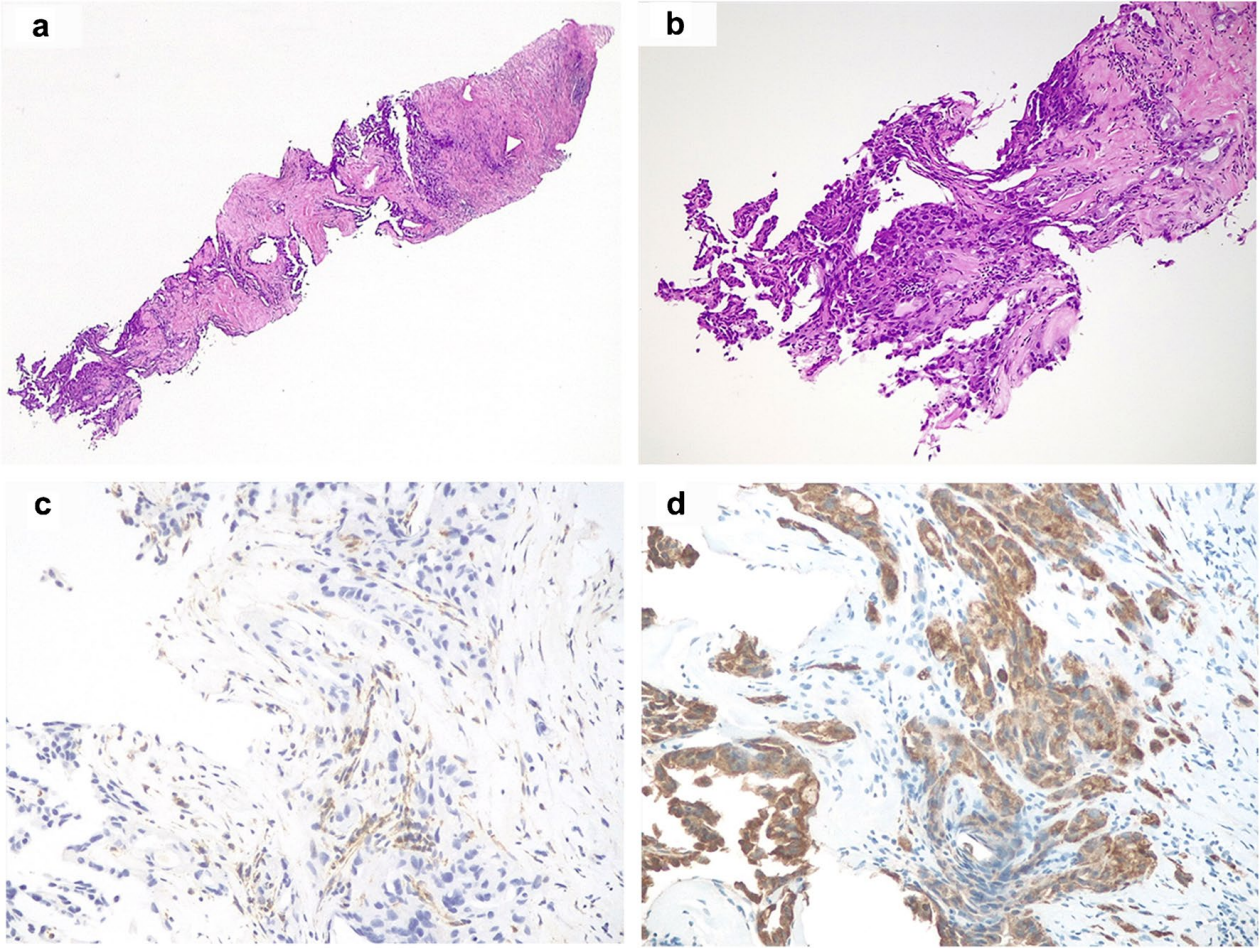
Hematoxylin and eosin microphotographs from the small renal biopsy of case 11 (**a**) showed a proliferation of high-grade eosinophilic cells embedded in a fibrous stroma (**b**). FH immunohistochemical loss (**c**) coupled with diffuse 2SC positivity (**d**) led to the diagnosis of FH-deficient renal cell carcinoma (original magnifications 25X (**a**), 100X (**b**), 200X (**c**), and (**d**))

A 41-year-old male patient was found with a 5-cm single solid lesion of the right kidney at CT scanning, along with secondary metastases involving multiple abdominal lymph nodes, the liver, and the lungs. He had already been diagnosed with a “hereditary leiomyomatosis and renal cell carcinoma syndrome” due to the previous discovery of an FH-deficient renal cell carcinoma in his father, deceased for the disease, and of uterine leiomyomas in a sister. Considering the ab initio metastatic condition, invasive major surgical resections were avoided. Thus, a core-needle biopsy of the renal mass was performed (Fig. 2), revealing a proliferation of high-grade neoplastic cells set in a fibrous stroma.

### Distribution of TILs and immunohistochemical findings

**Fig. 3:**
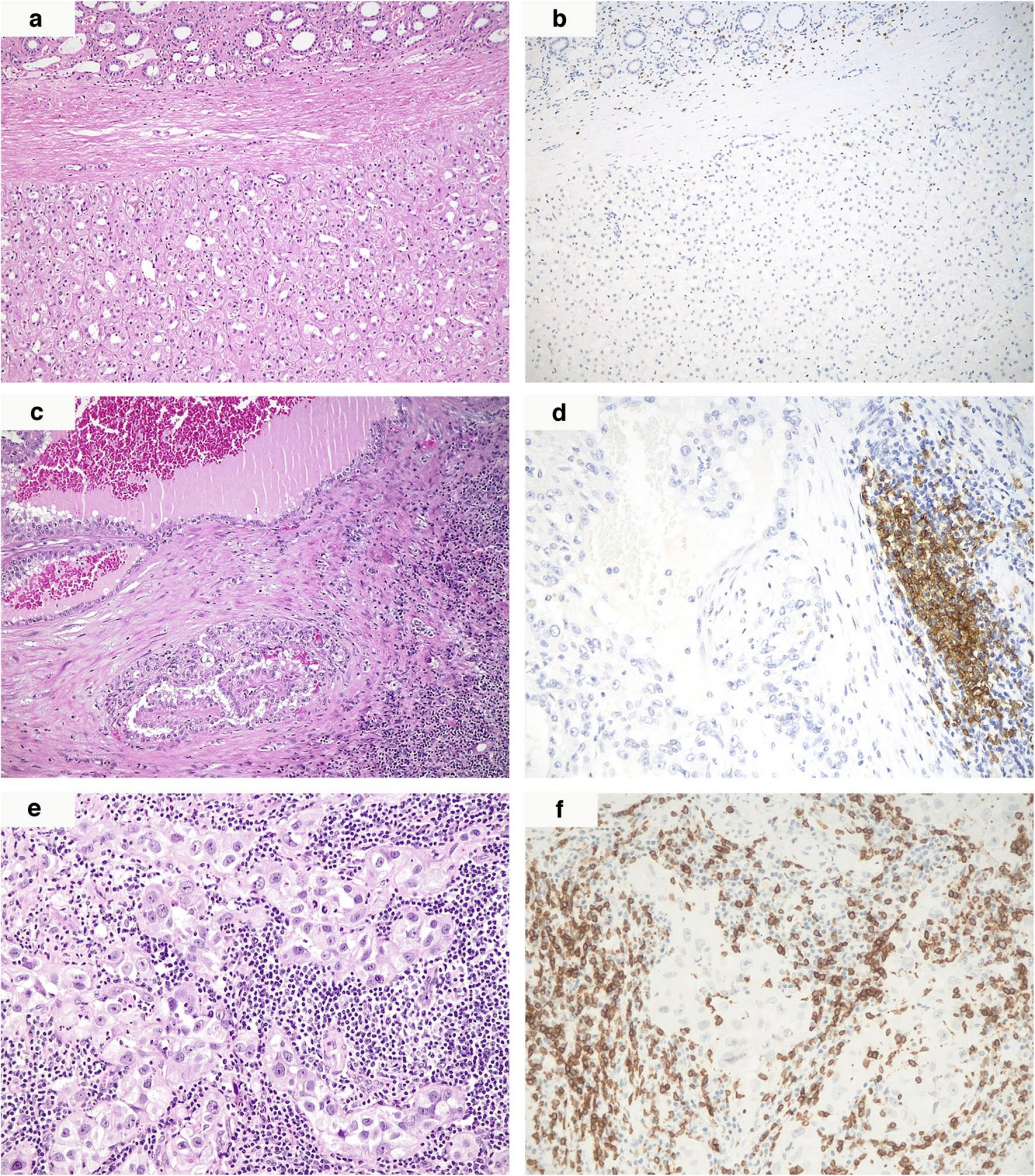
A “desert” sample (case 3) (**a**) with scattered if any CD3-positive T lymphocytes inside or outside the neoplastic area (**b**). “Excluded” neoplasms (case 2) were peripherally boarded by a mild inflammatory infiltrate (**c**), largely made up of CD3-positive T lymphocytes (**d**). In the only “inflamed” specimen of the series (case 9) (**e**), tumor cells were admixed with a remarkable CD3/CD8-positive T lymphocytic infiltrate (**f**) (original magnifications 100X (**a**–**c**), 200X (**d**–**f**)]

**Fig. 4:**
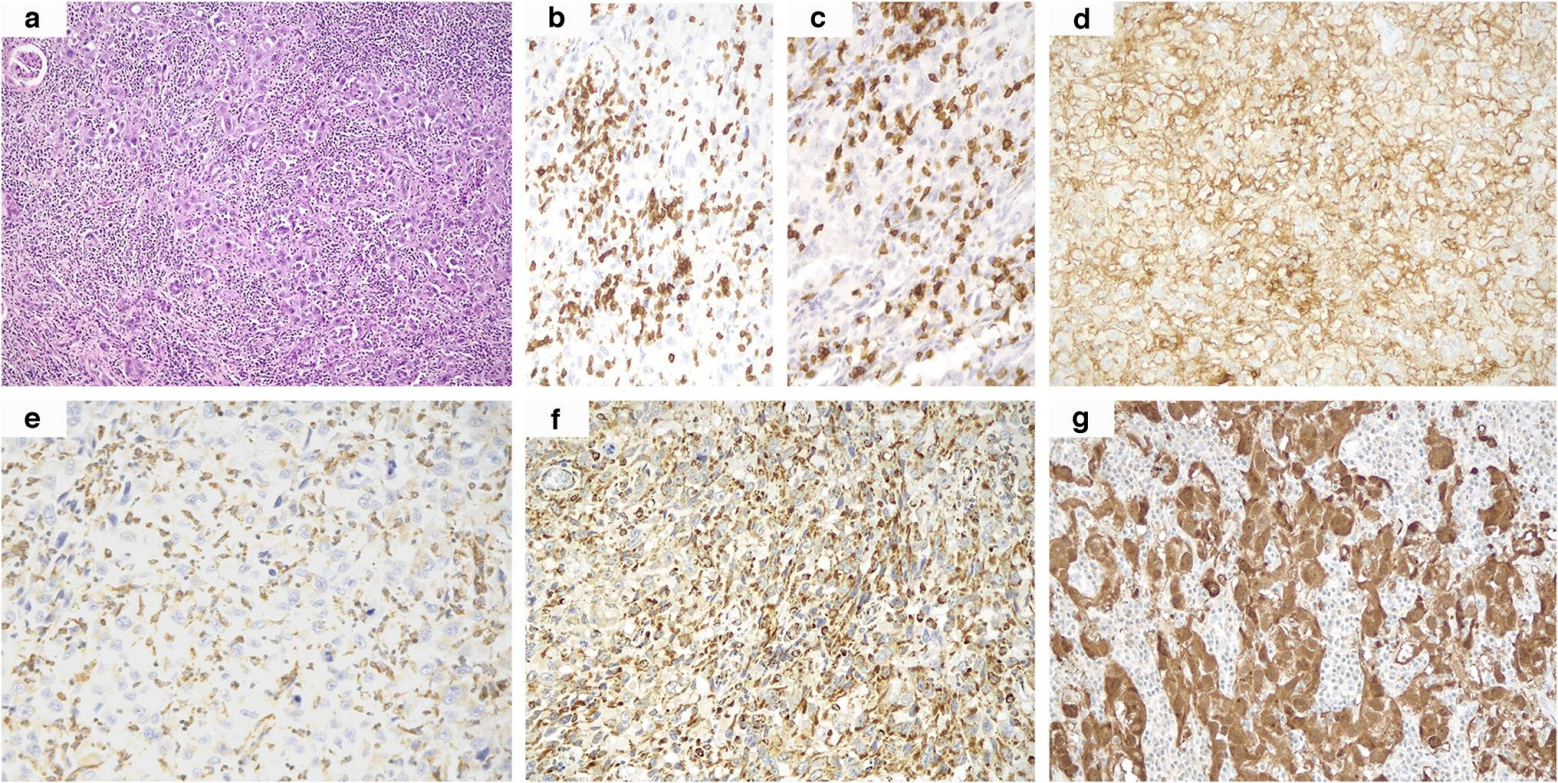
“Inflamed” distribution of TILs in case 9, with a marked lymphocytic infiltrate effacing neoplastic cells (**a**). TILs were mainly composed of CD3-positive (**b**) and CD8-positive (**c**) T-cytotoxic lymphocytes. Moreover, most tumor cells were stained for PD-L1 (**d**). While in some areas FH immunohistochemical loss could be easily assessed (**e**), in others the inflammatory infiltrate caused difficulty in its evaluation due to the prevalent amount of normally expressing immune cells (**f**). Conversely, 2SC immunohistochemistry was of great help in sorting the diagnostic quandary out, as it intensely and diffusely labeled the majority of neoplastic cells while not staining inflammatory elements (**g**) (original magnifications 100X (**a**), 200X (**b**–**g**))

TILs were evaluated in the whole series apart from the biopsy sample and were respectively scored as “desert” in 70% (7/10), “excluded” in 20% (2/10), or “inflamed” in 10% (1/10) of the specimens (Figs. 3a, c, e and 4a). Regarding the composition of TILs, the immunohistochemical analyses recalled the findings of our previous report, with both “excluded” and “inflamed” tumors mainly revealing a prevalent CD3 + CD8 + T-lymphocytic response (Figs. 3b, d, f and 4b, c) [5]. Similarly, regarding targetable immune-related markers, none of the tumor samples showed significant PD-1 immunolabeling in either the neoplastic cells or the inflammatory infiltrate. Conversely, a noteworthy proportion of the tumors tested (8/10, 80%) displayed at least focally positive PD-L1 immunohistochemical staining in their neoplastic elements, ranging from 2 to 70% of the cells (mean 15%, median 7.5%). It is worth mentioning that both the tumors showing the highest PD-L1 values, respectively, 30% (case 7) and 70% (case 9), displayed sarcomatoid dedifferentiation. Notably, the utmost PD-L1 immunohistochemical expression was detected in the entire series only “inflamed” tumor (patient 9, Fig. 4d).

All but one kidney tumor and metastatic localizations revealed diffuse FH immunohistochemical loss, apart from the primary renal neoplasm from case 10, which displayed a faint retained expression of such a marker in about 20% of the cells (Fig. 1d and inset). Moreover, in case 9, despite the fact that some areas could be confidently labeled as FH-negative, in others, the immune infiltrate’s marked effacement of the neoplastic cells made identifying FH loss a challenging task (Fig. 4e, f). Similarly, diffuse and intense cytoplasmic and nuclear 2SC staining was observed in all primary and secondary samples in the wide majority of neoplastic cells, ranging from 50 to 100%, including the doubtful cases 9 and 10 and the small biopsy from case 11 (Figs. 1e, 2d, and 4g).

**Fig. 5:**
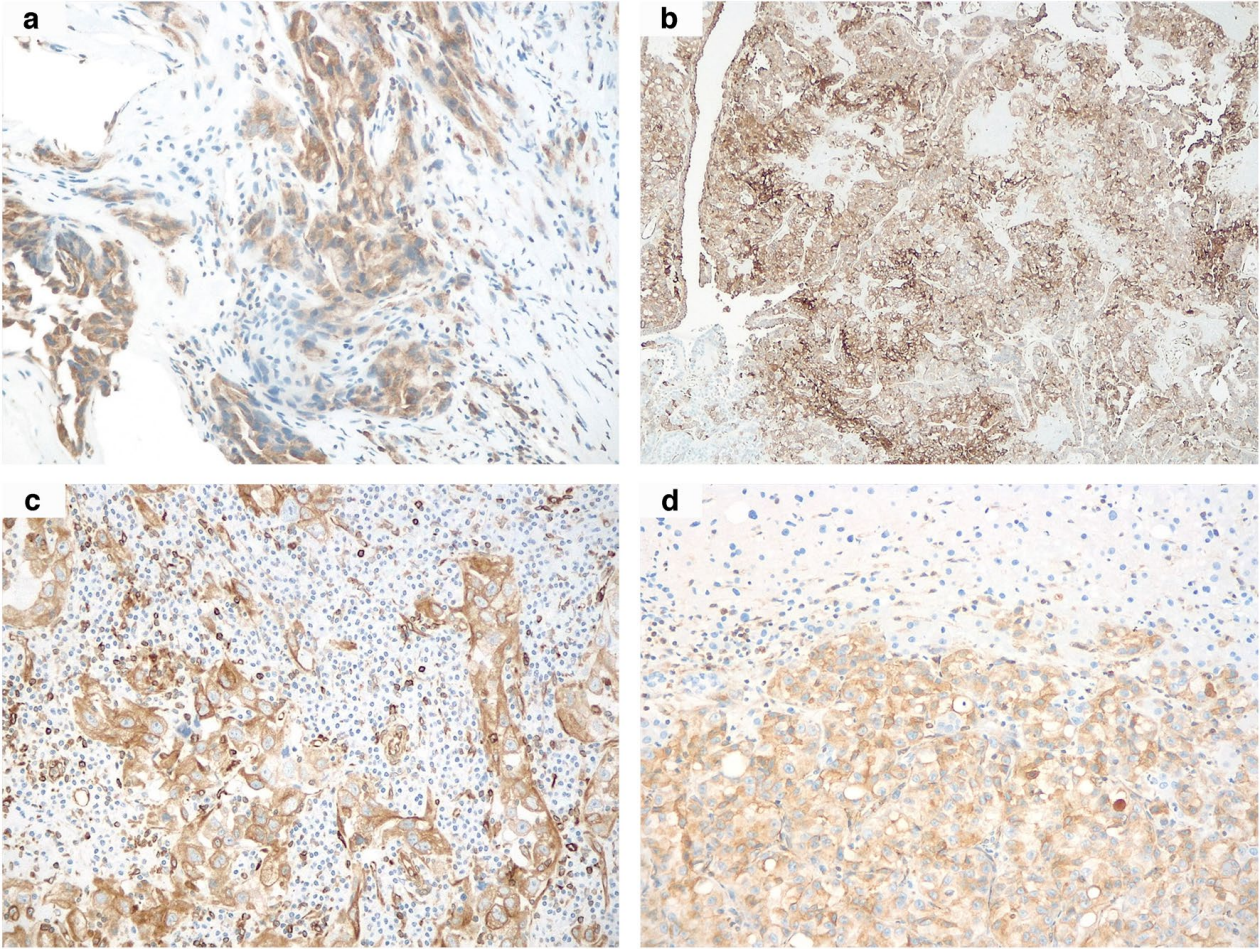
Strong and diffuse STING immunohistochemical expression was observed in the majority of the samples, including the small biopsy from case 11 (**a**), the partially FH-retaining case 10 (**b**), the inflamed case 9 (**c**), and the liver metastasis from case 6 (**d**) (original magnifications 100X (**b**), 200X (**a, c, d**))

Cytoplasmic STING immunostaining was recorded in most primary tumors (9/11, 82%), including the small biopsy from case 11 (Fig. 5a–c), ranging from 5 to 80% (mean 41%, median 40%), many of which (7/11, 78%) were in a wide percentage of neoplastic cells (≥ 30%). Interestingly, relevant STING labeling (≥ 30%) was observed in all but two aggressively behaving primary renal neoplasms, with one of the remaining tumors showing increased expression in the corresponding hepatic localization (case 6, 5% vs 40%, Fig. 5d). The other two histologically documented secondary localizations showed comparable STING expression rates with the primary renal neoplasm, respectively negative staining in the peritoneal metastasis from case 1 and 5% in the nodal metastasis from case 6. Apart from case 8, a relevant STING immunohistochemical positivity was detected in all primary renal tumors significantly labeling (≥ 5%) for PD-L1 (6/7, 86%). To note, the highest STING expression rate (80%) was observed in the primary kidney neoplasm displaying the utmost PD-L1 value (70%, case 9). As for PD-L1 negative samples, while one of them (case 1) also failed to label for STING, the other (case 4) was positive for such a marker in the majority of neoplastic cells (60%). Finally, as for the correlation with the distribution of TILs, it is worth mentioning that the maximum STING expression rate was observed in the only “inflamed” primary renal tumor (case 9), bearing the highest PD-L1 level as well (70%). Similarly, regarding the two “excluded” neoplasms instead, while one of them (case 2) showed high STING expression (70%) along with relevant PD-L1 staining (10%), the other (case 6) displayed low STING (5%) and PD-L1 levels (2%). However, as mentioned above, an increased STING staining was documented in the liver metastasis from the latter case compared to the primary tumor (40% vs 5%).

The clinical and pathological findings of primary renal tumors and secondary localizations of the present series are summarized in Table 1.

**Table 1:** Underlying pathogenic condition, immunohistochemical findings, distribution of tumor-infiltrating lymphocytes (TILs), and clinical outcomes of primary renal tumors and secondary localizations of FH-deficient renal cell carcinomas of the present series

Case no	Primary tumor						Clinical behavior (mo)	Metastasis			
	Sporadic/hereditary	FH	2SC	STING	TILs	PDL1 SP263		Site	FH	2SC	STING
1	NA^#^	Neg.^#^	100% +	Neg	Desert^#^	Neg	77 AWD	Peritoneum	Neg.^#^	100% +	Neg
2	Sporadic^#^	Neg.^#^	100% +	70% +	Excluded^#^	10% +	14 AWD				
3	NA^#^	Neg.^#^	100% +	5% +	Desert^#^	10% +	NED				
4	NA^#^	Neg.^#^	100% +	60% +	Desert^#^	Neg	53 AWD				
5	NA^#^	Neg.^#^	100% +	70% +	Desert^#^	5% +	26 AWD				
6*	Hereditary^#^	Neg.^#^	100% +	5% +	Excluded^#^	2% +	DOD	Lymph node	Neg.^#^	100% +	5% +
								Liver	Neg.^#^	100% +	40% +
7*	Hereditary^#^	Neg.^#^	100% +	30% +	Desert^#^	30% +	42 DOD				
8	NA^#^	Neg.^#^	90% +	Neg	Desert^#^	5% +					
9	Hereditary^#^	Neg.^#^	100% +	80% +	Inflamed^#^	70% +	10 NED				
10	Hereditary	20% +	50% +	50% +	Desert	15% +	14 AWD				
11	Hereditary	Neg	80% +	80% +	NA	NA	2 AWD				

*mo* months, *TILs* tumor-infiltrating lymphocytes, *FH* fumarate hydratase, *NA* not available, *AWD* alive with disease, *NED* no evidence of disease, *DOD* dead of disease

^*^Siblings

^#^Previously reported [5]

## Discussion

FH-deficient renal cell carcinomas are rare neoplasms, whose pathological diagnosis may be challenging at times, as they frequently show heterogeneous morphological features both in primary and secondary neoplasms and variable immunohistochemical profiles. However, it is pivotal to recognize these tumors as they may harbor a hereditary signature and usually display dismal clinical behavior without response to therapeutic options conventionally employed for advanced renal cell carcinomas [2]. In the present work, we have correlated the immunohistochemical expression of FH, 2SC, and STING with the pathological and phenotypical findings of a series of FH-deficient renal cell carcinomas, pointing out: (i) the rarity of such a neoplastic entity (0.2% of in-house renal cell carcinomas); (ii) the utility of the combination of FH loss and positive 2SC staining now commercially available in all primary and secondary tumors, supporting the safe adoption of this latter marker in daily practice; (iii) a significant STING labeling in most of the sample tested, especially in those showing a severe outcome and “hot” immune-related features, suggesting both a biological and a potential predictive role for such a marker in this renal cell carcinoma histotype.

Since their initial descriptions, several morphological features have been accustomed to FH-renal cell carcinoma, including prominent viral-inclusion-like eosinophilic nucleoli [2]. Nevertheless, most of these histological hints have proven to be unspecific, as they are often shared with other renal cell-derived tumors, with the most reliable finding still being considered the coexistence of multiple architectural growth patterns within the same tumor and its metastases [5, 27]. Concerning immunohistochemistry, FH protein loss of staining has been widely acknowledged as a reliable surrogate of the underlying molecular alteration [1, 6], although not bearing a 100% sensitivity. In this view, in the present work, we have demonstrated that the employment of FH immunostaining along with commercially available S2C testing (clone CRB2005017_3) is advisable when dealing with neoplasms suspicious for FH-deficient renal carcinoma, as these markers carry complementary sensitivity and specificity accuracy. Namely, the detection of diffuse 2SC expression allowed us to confidently classify one of the included cases as an FH-deficient renal cell carcinoma, despite showing partial retained FH staining in a percentage of its tumor cells. Such a finding is likely linked to some missense *FH* gene mutations causing alterations of the enzyme’s functional activity rather than its intrinsic structure so that its immunohistochemical expression may not be totally lost in such (yet unquantified) instances [28]. Therefore, by highlighting the accumulation of succinated proteins associated with the loss of FH functionality, a broad and intense 2SC staining may greatly help in categorizing such tumors. As witnessed by our example, the evidence of 2SC positivity was crucial to getting to the proper pathological diagnosis, which further warranted genetic counseling and testing. If we had not performed the 2SC test, the heterogenous FH expression pattern might have led us to render a prudent report of “unclassified renal cell carcinoma,” ultimately not catching a successively proven underlying hereditary condition (case 10). Similarly, the clear-cut identification of diffuse cytoplasmic and nuclear 2SC staining significantly eased the classification of another sample, where the proper evaluation of FH expression was inferred by the marked effacement of neoplastic cells by the inflammatory infiltrate (case 9). Indeed, it is worth mentioning that, despite harboring very high sensitivity rates, 2SC immunolabeling is not restricted to FH-deficient renal cell carcinoma at all, as other renal neoplasms may show variable cytoplasmic expression, especially papillary renal cell carcinoma [29]. Therefore, identifying both cytoplasmic and nuclear staining for such a marker is suggested to be considered reliable, alongside the evidence of its negative staining in the adjacent normal renal parenchyma. Ultimately, the combination of FH and 2SC immunohistochemical testing is more reliable than each marker all along, so it is advisable to perform both assays in tumors with clinical and pathological features suspicious for FH-deficient renal cell carcinoma [12]. In this view, physicians can potentially identify all tumors belonging to this entity thanks to two immunohistochemical tests, much more affordable than, indeed, accurate but certainly expansive genetic *FH* testing.

The strong and diffuse 2SC expression in FH-deficient renal cell carcinomas from our series is likely related to the accumulation of mitochondrial succinated proteins due to FH loss. According to previous preclinical study [17], this phenomenon may cause mitochondrial vesicles containing mtDNA to be conveyed to the cytoplasm, where they can trigger the cGAS-STING pathway. In our cohort, we have recorded significant STING immunohistochemical expression in the broad majority of primary renal tumors (80%) and two-thirds of metastatic specimens (67%) tested. Among renal neoplasms, such a marker has been recently tested in clear cell renal cell carcinoma [30], medullary renal cell carcinoma [31], TFE3-rearranged renal cell carcinoma, TFEB-altered renal cell carcinoma, and in the spectrum of perivascular epithelioid cell (PEC) lesions of the kidney [32]. As for the two studies relying on immunohistochemistry [30, 32], they employed the same antibody clone (anti-TMEM173; clone SP338, dilution 1:150; Abcam, UK). Regarding STING staining, the authors reported remarkable expression in high-grade aggressive clear cell renal cell carcinomas [30] and PEC lesions of the kidney [30], while TFE3/TFEB-rearranged renal cell carcinomas failed to label for this molecule. To the best of our knowledge, this is the first study to investigate STING immunostaining in a clinical series of FH-deficient renal cell carcinoma, claiming both a pivotal biological and predictive role for such a molecule in these tumors. In detail, it is indeed worth noting that relevant STING immunolabeling (≥ 30%) was observed in three to four (6/8, 75%) primary renal tumors developing adverse clinical behavior, with a further negative case displaying increased STING staining in a liver metastasis compared to the negative primary tumor. Such data compare with known evidence of strong STING expression in other aggressive renal cell neoplasms, including advanced clear cell renal cell carcinoma [30] and medullary renal cell carcinoma [31]. Although the precise underlying pathogenic bases are far from being recognized, it could be tempting to speculate that chronic inflammatory response might be linked to the role of STING in favoring tumor progression in FH-deficient renal cell carcinoma. Namely, STING is a well-known trigger of the interferon-mediated inflammatory reaction, and a discrete immune reaction has been previously described in several examples of FH-deficient renal cell carcinoma [24]. In this view, the aforementioned preclinical studies have shown that murine FH-deficient renal cell carcinoma models harbor high levels of IL-6 [17]. Previous works on triple-negative breast cancer lines have demonstrated that STING can stimulate an IL-6 response, which may cause cancer cells to survive [33]. As increased IL-6 levels have been associated with poor clinical outcomes in renal cell carcinoma patients [34], these data all suggest that STING expression could highlight the biological aggressiveness.

Finally, the evidence of a relevant role for STING in aggressive FH-deficient renal cell carcinomas may also harbor remarkable therapeutic implications. Nowadays, despite some trials being conducted testing VEGFR or EGFR inhibitors [35, 36], no standard therapy has been approved for FH-deficient renal cell carcinoma patients. However, a subset of these patients has been found to display significant PD-L1 levels, ranging from 58 to 69% of the samples in different cohorts [37, 38]. Our series’ data align with previous reports, with 70% of primary renal tumors showing relevant PD-L1 staining (≥ 5%). Interestingly, all but one of these neoplasms revealed at least focal STING cytoplasmic labeling (86%, 6/7). Similarly, the highest STING and PD-L1 rates were observed in the sample showing the most prominent inflammatory infiltrate, along with another “excluded” neoplasm diffusely labeling for STING and significantly for PD-L1. Indeed, due to the limited number of cases, further dedicated studies are warranted to investigate the link between STING and immune-related markers. Nevertheless, the blockage of the STING pathway in experimental models prevents local progression and metastasis development, eliciting a stronger antitumoral response [39]. Thus, it could be questioned whether the employment of STING antagonists could modulate PD-L1 expression and, therefore, the action of current immune checkpoint inhibitors targeting this molecule. These considerations notwithstanding, in the current hardly desert landscape of efficient therapeutic options for FH-deficient renal cell carcinoma, our work supports the adoption of STING as a potential predictive biomarker linked to response to immunotherapy regimens.

In conclusion, FH-deficient renal cell carcinomas are rare, heterogeneous, but highly aggressive neoplasms driven by somatic or germline mutations of the *FH* gene, which, among several pathways, trigger the c-GAS-STING signaling likely mediated by succination of mitochondrial proteins. In the present work, we have demonstrated a significant expression of STING in a vast proportion of FH-deficient renal cell carcinoma samples. As it was associated with aggressive clinical behavior and immune-related pathological findings, like the distribution of TILs and levels of PD-L1, we propose a potential predictive role for such a molecule. Moreover, the critical role of the succination process in the tumorigenesis of this neoplasm stresses the importance of testing both FH and 2SC to reach the proper pathological classification, preventing cases with challenging immunohistochemical features from being misdiagnosed. Our data further shed light on such a rare but almost invariably aggressive renal cell tumor, hopefully contributing to expanding the available knowledge on its pathogenesis to provide these patients with novel promising therapies.

## Data Availability

All data generated or analysed during this study are included in this published article.

## References

[1] Classification WHO, of Tumours Editorial Board, (eds) (2022) WHO classification of tumours, urinary and male genital tumours, 5th edn. IARC press, Lyon

[2] Merino MJ, Torres-Cabala C, Pinto P, Linehan WM (2007) The morphologic spectrum of kidney tumors in hereditary leiomyomatosis and renal cell carcinoma (HLRCC) syndrome. Am J Surg Pathol 31:1578–1585. 10.1097/PAS.0b013e31804375b8 17895761 10.1097/PAS.0b013e31804375b8

[3] Lau HD, Chan E, Fan AC, Kunder CA, Williamson SR, Zhou M, Idrees MT, Maclean FM, Gill AJ, Kao C-S (2020) A Clinicopathologic and molecular analysis of fumarate hydratase-deficient renal cell carcinoma in 32 patients. Am J Surg Pathol 44:98–110. 10.1097/PAS.0000000000001372 31524643 10.1097/PAS.0000000000001372

[4] Smith SC, Sirohi D, Ohe C, McHugh JB, Hornick JL, Kalariya J, Karia S, Snape K, Hodgson SV, Cani AK, Hovelson D, Luthringer DJ, Martignoni G, Chen Y-B, Tomlins SA, Mehra R, Amin MB (2017) A distinctive, low-grade oncocytic fumarate hydratase-deficient renal cell carcinoma, morphologically reminiscent of succinate dehydrogenase-deficient renal cell carcinoma. Histopathology 71:42–52. 10.1111/his.13183 28165631 10.1111/his.13183

[5] Caliò A, Marletta S, Stefanizzi L, Marcolini L, Rotellini M, Serio G, Bariani E, Vicentini C, Pedron S, Martelli FM, Antonini P, Brunelli M, Martignoni G (2024) Comparison of primary and metastatic fumarate hydratase-deficient renal cell carcinomas documents morphologic divergence and potential diagnostic pitfall with peritoneal mesothelioma. Mod Pathol 37:100561. 10.1016/j.modpat.2024.100561 38996839 10.1016/j.modpat.2024.100561

[6] Trpkov K, Hes O, Agaimy A, Bonert M, Martinek P, Magi-Galluzzi C, Kristiansen G, Lüders C, Nesi G, Compérat E, Sibony M, Berney DM, Mehra R, Brimo F, Hartmann A, Husain A, Frizzell N, Hills K, Maclean F, Srinivasan B, Gill AJ (2016) Fumarate hydratase-deficient renal cell carcinoma is strongly correlated with fumarate hydratase mutation and hereditary leiomyomatosis and renal cell carcinoma syndrome. Am J Surg Pathol 40:865–875. 10.1097/PAS.0000000000000617 26900816 10.1097/PAS.0000000000000617

[7] Shyu I, Mirsadraei L, Wang X, Robila V, Mehra R, McHugh JB, Chen Y-B, Udager AM, Gill AJ, Cheng L, Amin MB, Lin O, Smith SC (2018) Clues to recognition of fumarate hydratase-deficient renal cell carcinoma: findings from cytologic and limited biopsy samples. Cancer Cytopathol 126:992–1002. 10.1002/cncy.22071 30339328 10.1002/cncy.22071 PMC9116881

[8] Gleeson JP, Nikolovski I, Dinatale R, Zucker M, Knezevic A, Patil S, Ged Y, Kotecha RR, Shapnik N, Murray S, Russo P, Coleman J, Lee CH, Stadler ZK, Hakimi AA, Feldman DR, Motzer RJ, Reznik E, Voss MH, Chen Y-B, Carlo MI (2021) Comprehensive molecular characterization and response to therapy in fumarate hydratase-deficient renal cell carcinoma. Clin Cancer Res 27:2910–2919. 10.1158/1078-0432.CCR-20-4367 33658299 10.1158/1078-0432.CCR-20-4367 PMC8127353

[9] Pollard PJ, Brière JJ, Alam NA, Barwell J, Barclay E, Wortham NC, Hunt T, Mitchell M, Olpin S, Moat SJ, Hargreaves IP, Heales SJ, Chung YL, Griffiths JR, Dalgleish A, McGrath JA, Gleeson MJ, Hodgson SV, Poulsom R, Rustin P, Tomlinson IPM (2005) Accumulation of Krebs cycle intermediates and over-expression of HIF1alpha in tumours which result from germline FH and SDH mutations. Hum Mol Genet 14:2231–2239. 10.1093/hmg/ddi227 15987702 10.1093/hmg/ddi227

[10] Alderson NL, Wang Y, Blatnik M, Frizzell N, Walla MD, Lyons TJ, Alt N, Carson JA, Nagai R, Thorpe SR, Baynes JW (2006) S-(2-Succinyl)cysteine: a novel chemical modification of tissue proteins by a Krebs cycle intermediate. Arch Biochem Biophys 450:1–8. 10.1016/j.abb.2006.03.005 16624247 10.1016/j.abb.2006.03.005

[11] Bardella C, El-Bahrawy M, Frizzell N, Adam J, Ternette N, Hatipoglu E, Howarth K, O’Flaherty L, Roberts I, Turner G, Taylor J, Giaslakiotis K, Macaulay VM, Harris AL, Chandra A, Lehtonen HJ, Launonen V, Aaltonen LA, Pugh CW, Mihai R, Trudgian D, Kessler B, Baynes JW, Ratcliffe PJ, Tomlinson IP, Pollard PJ (2011) Aberrant succination of proteins in fumarate hydratase-deficient mice and HLRCC patients is a robust biomarker of mutation status. J Pathol 225:4–11. 10.1002/path.2932 21630274 10.1002/path.2932

[12] Mannan R, Wang X, Bawa PS, Chugh S, Chinnaiyan AK, Rangaswamy R, Zhang Y, Cao X, Smith SC, Trpkov K, Williamson SR, Sangoi AR, Mohanty S, McKenney JK, Gupta S, Magi-Galluzzi C, Argani P, Osunkoya AO, Chinnaiyan AM, Dhanasekaran SM, Mehra R (2023) Characterization of protein S-(2-succino)-cysteine (2SC) succination as a biomarker for fumarate hydratase-deficient renal cell carcinoma. Hum Pathol 134:102–113. 10.1016/j.humpath.2022.12.013 36581128 10.1016/j.humpath.2022.12.013

[13] Linehan WM, Rouault TA (2013) Molecular pathways: Fumarate hydratase-deficient kidney cancer–targeting the Warburg effect in cancer. Clin Cancer Res 19:3345–3352. 10.1158/1078-0432.CCR-13-0304 23633457 10.1158/1078-0432.CCR-13-0304 PMC4447120

[14] Peng X-H, Karna P, Cao Z, Jiang B-H, Zhou M, Yang L (2006) Cross-talk between epidermal growth factor receptor and hypoxia-inducible factor-1alpha signal pathways increases resistance to apoptosis by up-regulating survivin gene expression. J Biol Chem 281:25903–25914. 10.1074/jbc.M603414200 16847054 10.1074/jbc.M603414200 PMC3132567

[15] Naumov GN, Nilsson MB, Cascone T, Briggs A, Straume O, Akslen LA, Lifshits E, Byers LA, Xu L, Wu H-K, Jänne P, Kobayashi S, Halmos B, Tenen D, Tang XM, Engelman J, Yeap B, Folkman J, Johnson BE, Heymach JV (2009) Combined vascular endothelial growth factor receptor and epidermal growth factor receptor (EGFR) blockade inhibits tumor growth in xenograft models of EGFR inhibitor resistance. Clin Cancer Res 15:3484–3494. 10.1158/1078-0432.CCR-08-2904 19447865 10.1158/1078-0432.CCR-08-2904 PMC2893040

[16] Poor TA, Chandel NS (2023) Mitochondrial molecule controls inflammation. Nature 615:401–402. 10.1038/d41586-023-00596-y 36890308 10.1038/d41586-023-00596-y

[17] Zecchini V, Paupe V, Herranz-Montoya I, Janssen J, Wortel IMN, Morris JL, Ferguson A, Chowdury SR, Segarra-Mondejar M, Costa ASH, Pereira GC, Tronci L, Young T, Nikitopoulou E, Yang M, Bihary D, Caicci F, Nagashima S, Speed A, Bokea K, Baig Z, Samarajiwa S, Tran M, Mitchell T, Johnson M, Prudent J, Frezza C (2023) Fumarate induces vesicular release of mtDNA to drive innate immunity. Nature 615:499–506. 10.1038/s41586-023-05770-w 36890229 10.1038/s41586-023-05770-w PMC10017517

[18] Hooftman A, Peace CG, Ryan DG, Day EA, Yang M, McGettrick AF, Yin M, Montano EN, Huo L, Toller-Kawahisa JE, Zecchini V, Ryan TAJ, Bolado-Carrancio A, Casey AM, Prag HA, Costa ASH, De Los Santos G, Ishimori M, Wallace DJ, Venuturupalli S, Nikitopoulou E, Frizzell N, Johansson C, Von Kriegsheim A, Murphy MP, Jefferies C, Frezza C, O’Neill LAJ (2023) Macrophage fumarate hydratase restrains mtRNA-mediated interferon production. Nature 615:490–498. 10.1038/s41586-023-05720-6 36890227 10.1038/s41586-019-0000-0 PMC10411300

[19] Sugiura A, McLelland G-L, Fon EA, McBride HM (2014) A new pathway for mitochondrial quality control: mitochondrial-derived vesicles. EMBO J 33:2142–2156. 10.15252/embj.201488104 25107473 10.15252/embj.201488104 PMC4282503

[20] Gui X, Yang H, Li T, Tan X, Shi P, Li M, Du F, Chen ZJ (2019) Autophagy induction via STING trafficking is a primordial function of the cGAS pathway. Nature 567:262–266. 10.1038/s41586-019-1006-9 30842662 10.1038/s41586-019-1006-9 PMC9417302

[21] Ishikawa H, Ma Z, Barber GN (2009) STING regulates intracellular DNA-mediated, type I interferon-dependent innate immunity. Nature 461:788–792. 10.1038/nature08476 19776740 10.1038/nature08476 PMC4664154

[22] Janko C, Schorn C, Grossmayer GE, Frey B, Herrmann M, Gaipl US, Munoz LE (2008) Inflammatory clearance of apoptotic remnants in systemic lupus erythematosus (SLE). Autoimmun Rev 8:9–12. 10.1016/j.autrev.2008.07.015 18703173 10.1016/j.autrev.2008.07.015

[23] Wei B, Xu L, Guo W, Wang Y, Wu J, Li X, Cai X, Hu J, Wang M, Xu Q, Liu W, Gu Y (2021) SHP2-mediated inhibition of DNA repair contributes to cGAS-STING activation and chemotherapeutic sensitivity in colon cancer. Cancer Res:. 10.1158/0008-5472.CAN-20-3738 10.1158/0008-5472.CAN-20-3738 33820798

[24] Dong P, Zhang X, Peng Y, Zhang Y, Liu R, Li Y, Pan Q, Wei W, Guo S, Zhang Z, Han H, Zhou F, Liu Y, He L (2022) Genomic characteristics and single-cell profiles after immunotherapy in fumarate hydratase-deficient renal cell carcinoma. Clin. Cancer Res 28:4807–4819. 10.1158/1078-0432.CCR-22-1279 36074152 10.1158/1078-0432.CCR-22-1279

[25] Binnewies M, Roberts EW, Kersten K, Chan V, Fearon DF, Merad M, Coussens LM, Gabrilovich DI, Ostrand-Rosenberg S, Hedrick CC, Vonderheide RH, Pittet MJ, Jain RK, Zou W, Howcroft TK, Woodhouse EC, Weinberg RA, Krummel MF (2018) Understanding the tumor immune microenvironment (TIME) for effective therapy. Nat Med 24:541–550. 10.1038/s41591-018-0014-x 29686425 10.1038/s41591-018-0014-x PMC5998822

[26] Takahara T, Murase Y, Tsuzuki T (2021) Urothelial carcinoma: variant histology, molecular subtyping, and immunophenotyping significant for treatment outcomes. Pathology 53:56–66. 10.1016/j.pathol.2020.09.004 33070956 10.1016/j.pathol.2020.09.004

[27] Muller M, Guillaud-Bataille M, Salleron J, Genestie C, Deveaux S, Slama A, de Paillerets BB, Richard S, Benusiglio PR, Ferlicot S (2018) Pattern multiplicity and fumarate hydratase (FH)/S-(2-succino)-cysteine (2SC) staining but not eosinophilic nucleoli with perinucleolar halos differentiate hereditary leiomyomatosis and renal cell carcinoma-associated renal cell carcinomas from kidney tu. Mod Pathol 31:974–983. 10.1038/s41379-018-0017-7 29410489 10.1038/s41379-018-0017-7

[28] Skala SL, Dhanasekaran SM, Mehra R (2018) Hereditary Leiomyomatosis and Renal Cell Carcinoma Syndrome (HLRCC): A contemporary review and practical discussion of the differential diagnosis for HLRCC-associated renal cell carcinoma. Arch Pathol Lab Med 142:1202–1215. 10.5858/arpa.2018-0216-RA 30281371 10.5858/arpa.2018-0216-RA

[29] Chen Y-B, Brannon AR, Toubaji A, Dudas ME, Won HH, Al-Ahmadie HA, Fine SW, Gopalan A, Frizzell N, Voss MH, Russo P, Berger MF, Tickoo SK, Reuter VE (2014) Hereditary leiomyomatosis and renal cell carcinoma syndrome-associated renal cancer: recognition of the syndrome by pathologic features and the utility of detecting aberrant succination by immunohistochemistry. Am J Surg Pathol 38:627–637. 10.1097/PAS.0000000000000163 24441663 10.1097/PAS.0000000000000163 PMC3984629

[30] Marletta S, Caliò A, Bogina G, Rizzo M, Brunelli M, Pedron S, Marcolini L, Stefanizzi L, Gobbo S, Princiotta A, Porta C, Pecoraro A, Antonelli A, Martignoni G (2023) STING is a prognostic factor related to tumor necrosis, sarcomatoid dedifferentiation, and distant metastasis in clear cell renal cell carcinoma. Virchows Arch 483:87–96. 10.1007/s00428-023-03549-y 37120444 10.1007/s00428-023-03549-y PMC10326155

[31] Msaouel P, Malouf GG, Su X, Yao H, Tripathi DN, Soeung M, Gao J, Rao P, Coarfa C, Creighton CJ, Bertocchio J-P, Kunnimalaiyaan S, Multani AS, Blando J, He R, Shapiro DD, Perelli L, Srinivasan S, Carbone F, Pilié PG, Karki M, Seervai RNH, Vokshi BH, Lopez-Terrada D, Cheng EH, Tang X, Lu W, Wistuba II, Thompson TC, Davidson I, Giuliani V, Schlacher K, Carugo A, Heffernan TP, Sharma P, Karam JA, Wood CG, Walker CL, Genovese G, Tannir NM (2020) Comprehensive molecular characterization identifies distinct genomic and immune hallmarks of renal medullary carcinoma. Cancer Cell 37:720-734.e13. 10.1016/j.ccell.2020.04.002 32359397 10.1016/j.ccell.2020.04.002 PMC7288373

[32] Caliò A, Brunelli M, Gobbo S, Pedron S, Segala D, Argani P, Martignoni G (2021) Stimulator of interferon genes (STING) immunohistochemical expression in the spectrum of perivascular epithelioid cell (PEC) lesions of the kidney. Pathology:. 10.1016/j.pathol.2020.09.025 10.1016/j.pathol.2020.09.025 33461798

[33] Yan H, Lu W, Wang F (2023) The cGAS-STING pathway: a therapeutic target in chromosomally unstable cancers. Signal Transduct Target Ther 8:45. 10.1038/s41392-023-01328-4 36717545 10.1038/s41392-023-01328-4 PMC9886966

[34] Wang Y, Zhang Y (2020) Prognostic role of interleukin-6 in renal cell carcinoma: a meta-analysis. Clin Transl Oncol 22:835–843. 10.1007/s12094-019-02192-x 31410730 10.1007/s12094-019-02192-x

[35] Tomar S, Kashyap L, Kapoor A (2022) Fumarate hydratase-deficient renal cell carcinoma in extended remission with bevacizumab and erlotinib. Ecancermedicalscience 16:1404. 10.3332/ecancer.2022.1404 35919231 10.3332/ecancer.2022.1404 PMC9300402

[36] Srinivasan R, Gurram S, Al Harthy M, Singer EA, Sidana A, Shuch BM, Ball MW, Friend JC, Mac L, Purcell E, Vocke C, Kong HH, Cowen EW, Choyke PL, Malayeri AA, Long L, Shih JH, Merino MJ, Linehan WM (2020) Results from a phase II study of bevacizumab and erlotinib in subjects with advanced hereditary leiomyomatosis and renal cell cancer (HLRCC) or sporadic papillary renal cell cancer. J Clin Oncol 38:5004. 10.1200/JCO.2020.38.15_suppl.5004

[37] Alaghehbandan R, Stehlik J, Trpkov K, Magi-Galluzzi C, Condom Mundo E, Pane Foix M, Berney D, Sibony M, Suster S, Agaimy A, Montiel DP, Pivovarcikova K, Michalova K, Daum O, Ondic O, Rotterova P, Dusek M, Hora M, Michal M, Hes O (2017) Programmed death-1 (PD-1) receptor/PD-1 ligand (PD-L1) expression in fumarate hydratase-deficient renal cell carcinoma. Ann Diagn Pathol 29:17–22. 10.1016/j.anndiagpath.2017.04.007 28807336 10.1016/j.anndiagpath.2017.04.007

[38] Walter B, Gil S, Naizhen X, Kruhlak MJ, Linehan WM, Srinivasan R, Merino MJ (2020) Determination of the expression of PD-L1 in the morphologic spectrum of renal cell carcinoma. J Cancer 11:3596–3603. 10.7150/jca.35738 32284756 10.7150/jca.35738 PMC7150459

[39] Bakhoum SF, Ngo B, Laughney AM, Cavallo J-A, Murphy CJ, Ly P, Shah P, Sriram RK, Watkins TBK, Taunk NK, Duran M, Pauli C, Shaw C, Chadalavada K, Rajasekhar VK, Genovese G, Venkatesan S, Birkbak NJ, McGranahan N, Lundquist M, LaPlant Q, Healey JH, Elemento O, Chung CH, Lee NY, Imielenski M, Nanjangud G, Pe’er D, Cleveland DW, Powell SN, Lammerding J, Swanton C, Cantley LC (2018) Chromosomal instability drives metastasis through a cytosolic DNA response. Nature 553:467–472. 10.1038/nature25432 29342134 10.1038/nature25432 PMC5785464

